# miR-34c disrupts spermatogonial stem cell homeostasis in cryptorchid testes by targeting Nanos2

**DOI:** 10.1186/s12958-018-0417-z

**Published:** 2018-10-15

**Authors:** Zhenyu Huang, Dongdong Tang, Jingjing Gao, Xianming Dou, Peng Cheng, Dangwei Peng, Yao Zhang, Jun Mao, Li Zhang, Xiansheng Zhang

**Affiliations:** 1 Department of Urology, The First Affiliated Hospital of Anhui Medical University, Hefei, 230022 China; 2 Reproductive Medicine Center, Department of Obstetrics and Gynecology, The First Affiliated Hospital of Anhui Medical University, Hefei, 230022 China; 3 Anhui Province Key Laboratory of Reproductive Health and Genetics, Anhui Medical University, Hefei, 230032 China; 4 Anhui Provincial Engineering Technology Research Center for Biopreservation and Artificial Organs, Hefei, 230022 China

**Keywords:** Cryptorchidism, miR-34c, Nanos2, Spermatogonial stem cell

## Abstract

**Background:**

Cryptorchidism as a common genitourinary malformation with the serious complication of male infertility draws widespread attention. With several reported miRNAs playing critical roles in spermatogonial stem cells (SSCs), we aimed to explore the fundamental function of the highly conserved miR-34c in cryptorchidism.

**Methods:**

To explore whether miR-34c participates in spermatogenesis by regulating Nanos2, we examined the effect of overexpression and inhibition for miR-34c on Nanos2 expression in GC-1 cells. Moreover, the expression levels of miR-34c and Nanos2 with cryptorchidism in humans and mice were examined. Furthermore, the homeostasis of SSCs was evaluated through counting the number of promyelocytic leukemia zinc finger (PLZF) positive spermatogonia in murine cryptorchid testes.

**Results:**

In the present study, we show that miR-34c could inhibit the expression of Nanos2 in GC-1 cells. Meanwhile, miR-34c significantly decreased in both the testicular tissues of patients with cryptorchidism and surgery-induced murine model of cryptorchidism. Western blot revealed that the protein level of Nanos2 was up-regulated and showed to be negatively correlated to the expression of miR-34c in our model. The abnormal expression of miR-34c/Nanos2 disrupted the balance between SSC self-renewal and differentiation, eventually damaging the spermatogenesis of cryptorchid testes.

**Conclusions:**

The miR-34c/Nanos2 pathway provides new insight into the mechanism of male infertility caused by cryptorchidism. Our results indicate that miR-34c may serve as a biological marker for treatment of infertility caused by cryptorchidism.

## Background

Male infertility is a serious health problem that has been receiving more and more attention around the world [1, 2]. Idiopathic diseases of the male reproductive system, such as cryptorchidism and varicocele, are important factors that lead to male infertility [3, 4]. As a common genitourinary malformation, cryptorchidism is a condition that occurs due the continual exposure of the undescended testis to elevated temperatures because of its location in the abdominal cavity or canalis inguinalis, culminating in serious damage to spermatogenesis. Spermatogonial stem cells (SSCs), which possess self-renewal capacity and could differentiate into spermatozoa, are the foundation of spermatogenesis [5]. The balance between self-renewal and differentiation in SSCs ensures stable and persistent spermatogenesis in the testes. Nanos2, an evolutionarily conserved RNA binding protein, is the marker of undifferentiated spermatogonia and is found to be almost exclusively expressed in A_s_ to A_pr_ subpopulation of spermatogonia [6]. Recent studies have reported that Nanos2-mediated post-transcriptional regulation is essential for homeostasis of murine SSCs. Nanos2 maintains SSC homeostasis by the translational repression of differentiation-related mRNAs and the sequestration of the mTOR protein [7]. In contrast, highly expressed Nanos2 resulted in the accumulation of promyelocytic leukemia zinc finger (PLZF) positive spermatogonia and the destruction of SSC balance [8].

MicroRNAs (miRNAs), small non-coding RNA molecules of ~ 22 nucleotides, target the 3′-untranslated region (3’UTR) of mRNAs to regulate protein expression in various biological processes [9]. A number of miRNAs, such as miR-17, miR-18, miR-19 and miR-25, have been demonstrated to play critical roles in spermatogenesis. They contribute to early spermatogonial differentiation and deficiency of the miR-34 family leads to mouse infertility [10]. miR-34c, belonging to the highly conserved miR-34 family across diverse species, was reported to be associated with proliferation, apoptosis and invasion of multiple malignancies, such as breast cancer and endometrial carcinoma [11–13]. Moreover, miR-34c is preferentially expressed in the testes of mice and participates in spermatogenesis by regulating targets. For example, miR-34c enhances male germ cell apoptosis through targeting ATF1 and regulates murine testicular development and spermatogenesis by targeting the E2F-pRb pathway [14, 15]. Furthermore, simultaneous inactivation of miR-34b/c and miR-449 loci resulted in oligoasthenoteratozoospermia and infertility in male mice [16, 17]. A previous study showed that miR-34c was abundantly expressed in SSC-enriched germ cell cultures and could enhance SSC differentiation [18]. Although the function of miR-34c was largely explored in multiple malignancies and SSCs, whether miR-34c is involved in congenital genitourinary malformations, particularly cryptorchidism, remains unclear.

To address this question, we checked the expression level of miR-34c in cryptorchidism and explore its underlying mechanisms. We found that miR-34c was significantly decreased in the testicular tissues of patients with cryptorchidism. Moreover, the downregulation of miR-34c increases the protein level of Nanos2 to disrupt SSC homeostasis in murine cryptorchid testes. The miR-34c/Nanos2 pathway may provide new insight into the mechanism of male infertility caused by cryptorchidism.

## Methods

### Human tissue samples

Human testicular tissues were obtained from the First Affiliated Hospital of Anhui Medical University (Hefei, China). All patients, whose ages ranged from 18 to 33 years old, signed an informed consent, and their tissues were allowed for research purposes. Six samples of testicular tissue from patients with cryptorchidism and another six samples of testicular tissue from patients with male infertility caused by obstructive azoospermia (OA) were collected for comparison. This study was approved by the Ethics Committee of the First Affiliated Hospital of Anhui Medical University.

### Cell culture, plasmid construction and transfection

GC-1 cell lines were obtained from BeNa Culture Collection (Bncc Biotech, Beijing, China) and maintained according to their recommendations. The plasmid for overexpressing mouse miR-34c was constructed with recombinant methods (Vazyme c113–02). Firstly, the vector named pmR-mCherry (ClonTech) was linearized with EcoRI and BamHI. Then the amplified fragment was purified and inserted into the backbone. Primer sequences were as followed: forward: 5’-CCGGAAACTAGTCTCAGATCTGCTGTGTGGTTAGTGATTGG-3′ and reverse: 5’-GATTATGATCAGTTATCTAGATACCTGTGTGTAAGAGCCAG-3′. The miR-34c inhibitor was synthesized by Ribobio (Ribobio Biotech, Guangzhou, China). Plasmid transfection was conducted with Lipofectamine 2000 (Invitrogen) according to the manufacturer’s protocol with G418 selection (400 μg/mL). Transfection of RNA inhibitor was conducted with Oligofectamine (Invitrogen) according to the manufacturer’s protocol.

### Animals

All mice were handled in accordance with the relevant guidelines, and all experimental procedures were approved by the Ethics Committee of Anhui Medical University. A total of 60 male ICR mice, 6-weeks-old, were obtained from the Laboratory Animal Centre of Anhui Medical University and kept in a specific pathogen-free animal facility. The mice were exposed to a 14 h light/10 h dark cycle and maintained at a suitable temperature of 22 ± 2 °C with free access to food and water.

### Mouse model of cryptorchidism

Male ICR mice were randomly divided into two groups: the cryptorchidism group (*n* = 30) and the control group (*n* = 30). The skin of the left lower abdomen was disinfected after the mice were anaesthetized. In the cryptorchidism group, the left gubernaculum was sheared after opening the abdominal cavity, followed by suturing of the fat pad near the testes with peritoneum to fix the left testes in the abdominal cavity. In the control group, after opening the abdominal cavity, the left testes were gently pulled and left in the original scrotal position. No treatments were performed on the right testes in both groups. Mice were sacrificed by cervical dislocation at 3, 7, 14, 21 and 28 days after the operation was completed. The testes were obtained, then photographed and weighed.

### Histological analysis and immunohistochemistry

Testicular tissues were fixed with 4% paraformaldehyde, then embedded in paraffin and divide into 4-μm sections. To evaluate the histological changes of cryptorchid testes, the sections were stained with hematoxylin and eosin (HE)following a standard protocol. Furthermore, the location and expression of PLZF were determined by immunohistochemistry to assess the changes in the number of spermatogonia. The testicular tissue sections were heated in sodium citrate buffer (pH 6.0) for 2 mins and then dipped in deionized water containing 3% H2O2 for 20 mins to quench endogenous peroxidase activity. Next, the sections were incubated with PLZF-specific antibody (1:20; Santa Cruz Biotech, Dallas, TX, USA) at 4 °C overnight and then incubated with horseradish peroxidase-labelled secondary antibody at 37 °C for 30 mins. Finally, the sections were stained with diaminobenzidine and counterstained with Harris’s hematoxylin.

### Real-time RT-PCR

Total RNA was extracted from testicular tissues with TRIzol reagent (Invitrogen, Thermo Fisher Scientific, Inc., Foster City, CA, USA) following the manufacturer’s protocol. Complementary DNA was synthesized from RNA with the FastQuant RT Kit (Tiangen Biotech, Beijing, China) according to the manufacturer’s protocol. The primer of miR-34c used for cDNA synthesis was GTCGTATCCAGTGCAGGGTCCGAGGTATTCGCACTGGATACGACGCAATC. Real-time PCRs were performed with the SuperReal PreMix Plus (SYBR Green) Kit in an ABI7500 Real Time PCR System (Applied Biosystems; Thermo Fisher Scientific, Inc.). In addition, melting-curve analysis was used to monitor the purity of the PCR product. The expression levels of mature miR-34c and Nanos2 mRNA were normalized to 18S rRNA. The primer sequences are listed in Table 1.

**Table 1:** The primer sequences used for Real-time RT-PCR

Gene	Species	Forward	Reverse
Nanos2	Mouse	CCATATGCAACTTCTGCAA	ACTGCTGACTGCTGTTGAGTG
Nanos2	Human	CTGAGAAGTGCCTACTCAAG	TGATACGGTGCTCTCCAGAG
miR-34c	Human/Mouse	CACGCAAGGCAGTGTAGT	CCAGTGCAGGGTCCGAGGTA
18S	Human/Mouse	CGGCGACGACCCATTCGAAC	GAATCGAACCCTGATTCCCCGTC

### Western blotting

For preparation of samples, protein extracted from testicular tissues was minced and lysed in RIPA buffer (Beyotime Biotech, Shanghai, China) on ice more than 20 mins. After sonication, the concentrations were measured with a BCA protein assay kit (Beyotime Biotech). Total protein was loaded onto 15% SDS-PAGE gels and then transferred to PVDF membranes. Membranes were processed according to the ECL Western blotting protocol (GE Healthcare, General Electric Company, Boston, USA). Quantification of the band intensities was performed with ImageJ (NIH, Bethesda, MD, USA). An area above each band, the same size as the corresponding band, was used for background subtraction. The following antibodies were used in Western blots: anti-Nanos2 (1:200; GeneTex, Irvine, CA, USA) and anti-GAPDH (1:1000; TransGen Biotech, Beijing, China).

### Statistical analyses

The values shown in the graphs represent averages of at least three independent experiments, with error bars showing standard deviations. Two-sample Student’s t tests and Wilcoxon rank sum tests were carried out using SPSS version 22.0 (SPSS, Chicago, IL, USA). *P* < 0.05 was considered significantly different.

## Results

### miR-34c decreases in the testicular tissues of patients with cryptorchidism

**Fig. 1:**
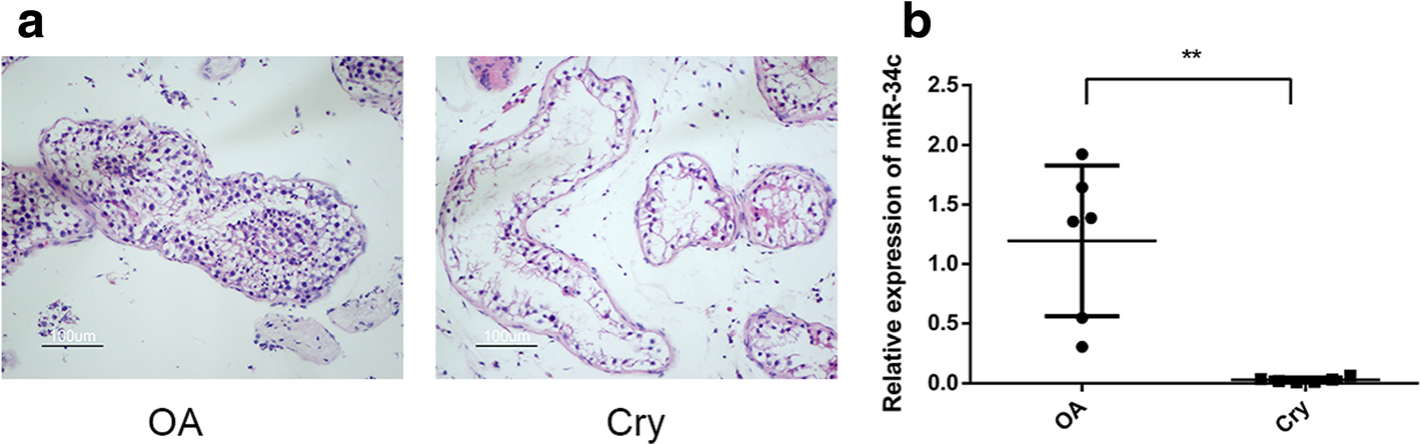
miR-34c was markedly reduced in the testicular tissues of patients with cryptorchidism. **a** The histological comparison of testicular tissues between cryptorchidism patients and OA patients. **b** The expression level of miR-34c in the testicular tissues of patients with cryptorchidism. OA patients were the control. Bar =100 μm. OA: obstructive azoospermia; Cry: cryptorchidism. ^**^P < 0.01

We investigated whether or not miR-34c functioned in the testicular tissues of patients with cryptorchidism. Six samples of testicular tissue from patients with cryptorchidism and another six samples of testicular tissue from patients with OA were used for comparison. Histological examination showed significant difference between the two groups (Fig. 1a). The Johnsen score was performed to evaluate the spermatogenesis of testes. The Johnsen scores of patients with cryptorchidism were just 2–3 scores (data not shown). Per real-time PCR, the expression level of miR-34c significantly decreased in the testicular tissues of patients with cryptorchidism compared to that of patients with OA (1.19 ± 0.633 vs 0.0292 ± 0.0207, *P* = 0.006) (Fig. 1b).

### miR-34c inhibits Nanos2 expression in GC-1 cells

**Fig. 2:**
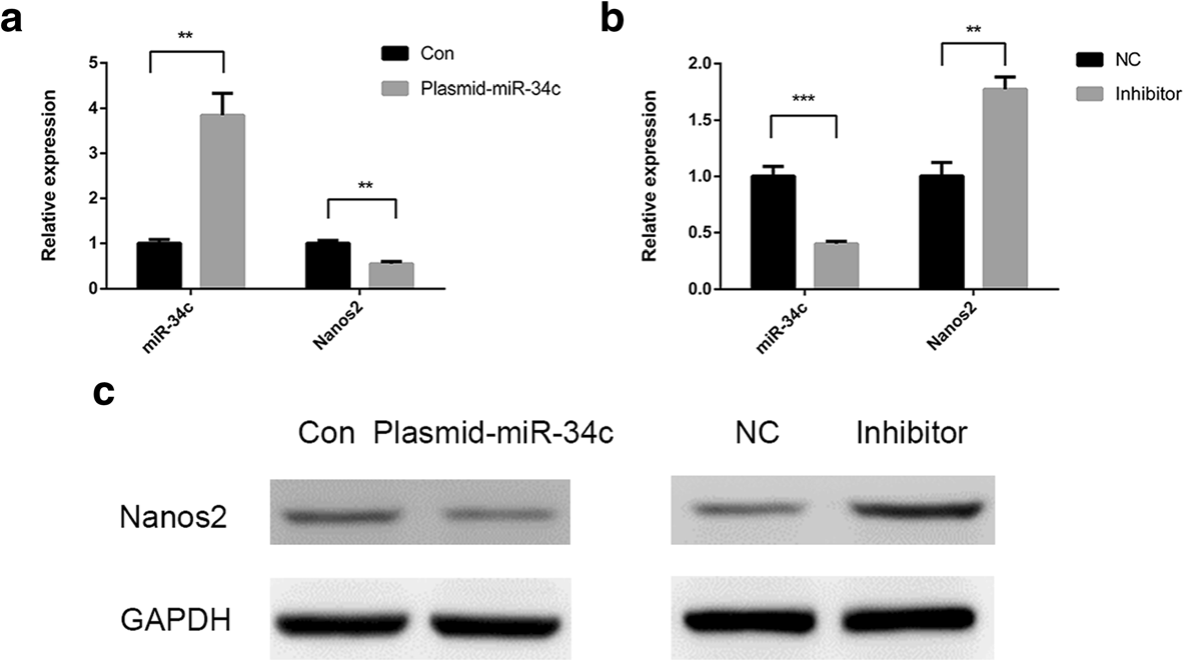
miR-34c inhibit the expression of Nanos2 in GC-1 cells. **a** Relative expression levels of miR-34c and Nanos2 mRNA were analyzed by RT-qPCR after transfecting the plasmid of miR-34c in GC-1 cell. **b** Relative expression levels of miR-34c and Nanos2 mRNA were analyzed by RT-qPCR after miR-34c inhibition in GC-1 cell. **c** Nanos2 protein levels in a GC-1 cell culture after miR-34c overexpression or inhibition. Con: control; NC: negative control. ^*^P < 0.05, ^**^P < 0.01 and ^***^P < 0.001

To investigate the roles of miR-34c in cryptorchidism, we focused on its mechanism of function. Using online prediction tools, such as TargetScan and miRDB, we found several targets of miR-34c, among which was Nanos2, a gene with great significance in SSC homeostasis. To explore whether miR-34c participates in spermatogenesis by regulating Nanos2, we examined the effect of overexpression and inhibition for miR-34c on Nanos2 expression in GC-1 cells. After transfecting the plasmid of miR-34c for 48 h in GC-1 cell, RT-qPCR showed the markedly increase of miR-34c level. RT-qPCR and western blot analysis demonstrated significant decrease of Nanos2 expression level (1.00 ± 0.0830 vs 3.85 ± 0.486, *P* = 0.008; 1.00 ± 0.0642 vs 0.556 ± 0.0483, *P* = 0.001) (Fig. 2a, c). In reverse, when treated with the inhibitor of miR-34c, GC-1 cells showed the down-regulation of miR-34c and up-regulation of Nanos2 expression level (1.00 ± 0.0871 vs 0.401 ± 0.0208, *P* < 0.001; 1.00 ± 0.119 vs 1.78 ± 0.105, *P* = 0.001) (Fig. 2b, c). All the results indicate that miR-34c could inhibit the expression of Nanos2 in GC-1 cells.

### Establishment of mouse model of cryptorchidism

**Fig. 3:**
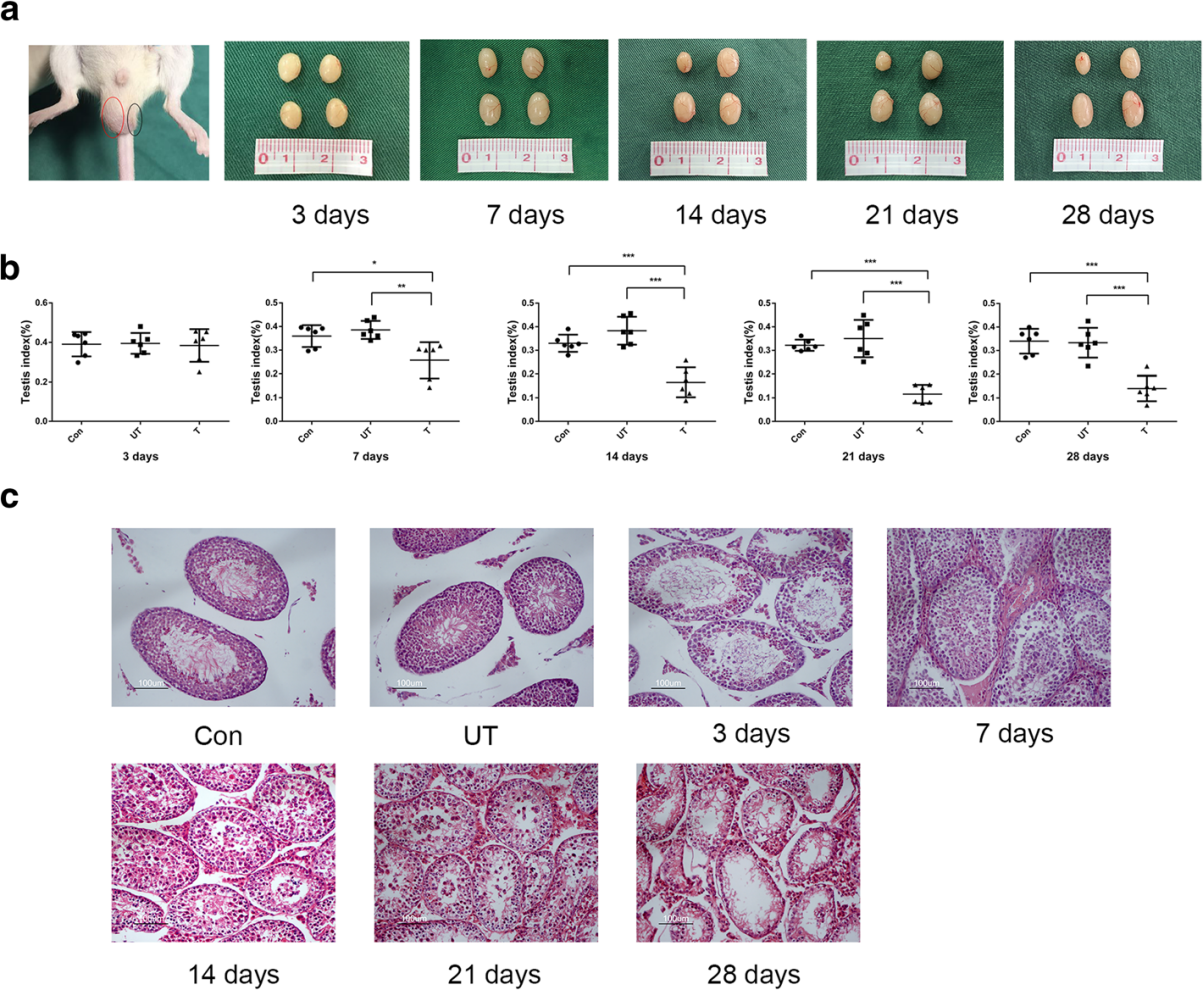
Mouse model of cryptorchidism. **a** The normal (red circle) and cryptorchid (black circle) mouse testes. Photograph of testes of cryptorchidism group (first row) and control group (second row). **b** The testis index of cryptorchidism group and control group. Testis index = (testis weight/whole body weight) * 100%. **c** The histological comparison of testicular tissues between cryptorchidism group and control group. Bar = 100 μm. Con: control; UT: untreated side; T: treated side. ^*^P < 0.05, ^**^P < 0.01 and ^***^P < 0.001

To further explore the role of miR-34c in cryptorchidism, we established a surgery-induced mouse model of cryptorchidism, as previously described [19, 20]. In the cryptorchidism group, the left testes, known as the treated side testes, of mice were fixed in the abdominal cavity to simulate an environment of cryptorchidism and the contralateral ones as the untreated side testes, serving as self-control. We also performed the operation on the left lower abdomen to exclude the influence of surgical trauma as group control. The contrast of testes between the cryptorchidism group and control group for 3, 7, 14, 21 and 28 days after operation are shown in Fig. 2a. Furthermore, the weight percentage of testes compared to the whole body was calculated as the testis index to evaluate the difference of testes of the two groups. We found that the treated side testes of the cryptorchidism group significantly decreased both in volume and in weight compared with the untreated side testes and control group at 7, 14, 21 and 28 days after operation (3 days: Con vs *T* = 0.390 ± 0.0617 vs 0.384 ± 0.0829, *P* = 0.882; UT vs *T* = 0.394 ± 0.0529 vs 0.384 ± 0.0829, *P* = 0.795. 7 days: Con vs *T* = 0.359 ± 0.0461 vs 0.258 ± 0.0761, *P* = 0.019; UT vs *T* = 0.386 ± 0.0385 vs 0.258 ± 0.0761, *P* = 0.007. 14 days: Con vs *T* = 0.330 ± 0.0364 vs 0.165 ± 0.0629, *P* < 0.001; UT vs *T* = 0.383 ± 0.0591 vs 0.165 ± 0.0629, *P* < 0.001. 21 days: Con vs *T* = 0.322 ± 0.0237 vs 0.116 ± 0.0389, *P* < 0.001; UT vs *T* = 0.350 ± 0.0792 vs 0.116 ± 0.0389, *P* < 0.001. 28 days: Con vs *T* = 0.340 ± 0.527 vs 0.140 ± 0.0539, *P* < 0.001; UT vs *T* = 0.333 ± 0.0632 vs 0.140 ± 0.0539, *P* < 0.001) (Fig. 3a, b). In addition, HE staining demonstrated the morphological changes of cryptorchid testes (Fig. 3c). The treated side testes retained normal spermatogenesis and showed no significant difference compared to the controls at 3 days after operation. However, cryptorchid testes showed obvious morphological abnormalities of seminiferous tubules at 7 days. From 14 to 28 days, we found that germ cells were progressively reduced and there was vacuolization in the seminiferous tubules of cryptorchid testes. At 28 days, only a small portion of germ cells remained in the basal part of the seminiferous tubules.

### miR-34c up-regulated Nanos2 in mouse model of cryptorchidism

**Fig. 4:**
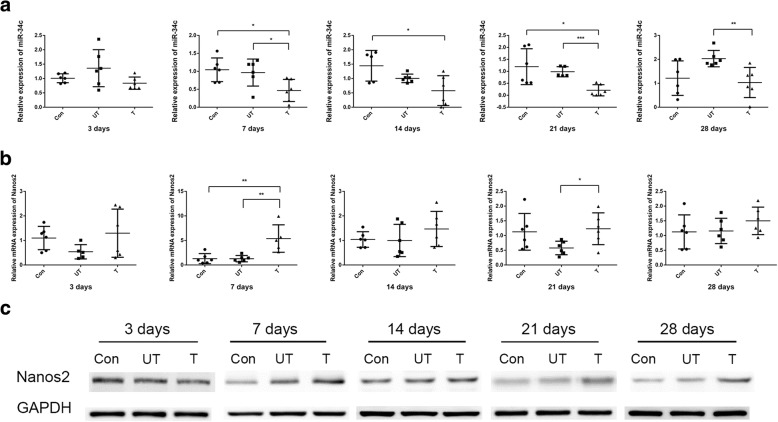
Nanos2 was upregulated by miR-34c in mouse model of cryptorchidism. **a** The expression level of miR-34c performed in treated side testes of cryptorchidism group compared to the untreated side testes and control group. **b** The mRNA level of Nanos2 performed in treated side testes of cryptorchidism group compared to the untreated side testes and control group. **c** The protein level of Nanos2 performed in treated side testes of cryptorchidism group compared to the untreated side testes and control group by western blot. Con: control; UT: untreated side; T: treated side; GAPDH: glyceraldehyde-3-phosphate dehydrogenase. ^*^P < 0.05, ^**^P < 0.01 and ^***^P < 0.001

Similar to the expression pattern in humans, we found that the miR-34c level was significantly down-regulated in the treated side testes (3 days: Con vs *T* = 1.01 ± 0.154 vs 0.837 ± 0.214, *P* = 0.139; UT vs *T* = 1.36 ± 0.643 vs 0.837 ± 0.214, *P* = 0.089. 7 days: Con vs *T* = 1.04 ± 0.330 vs 0.467 ± 0.308, *P* = 0.011; UT vs *T* = 0.966 ± 0.376 vs 0.467 ± 0.308, *P* = 0.031. 14 days: Con vs *T* = 1.44 ± 0.534 vs 0.577 ± 0.519, *P* = 0.024; UT vs T = 1.01 ± 0.148 vs 0.577 ± 0.519, *P* = 0.101. 21 days: Con vs *T* = 1.20 ± 0.750 vs 0.210 ± 0.234, *P* = 0.022; UT vs *T* = 0.984 ± 0.211 vs 0.210 ± 0.234, *P* < 0.001. 28 days: Con vs *T* = 1.21 ± 0.721 vs 1.03 ± 0.628, *P* = 0.625; UT vs *T* = 2.03 ± 0.343 vs 1.03 ± 0.628, *P* = 0.007) (Fig. 4a). The mRNA level of Nanos2 was significantly up-regulated in the treated side testes of the cryptorchidism group at 7 and 21 days (3 days: Con vs *T* = 1.10 ± 0.476 vs 1.30 ± 0.981, *P* = 0.671; UT vs *T* = 0.538 ± 0.291 vs 1.30 ± 0.981, *P* = 0.122. 7 days: Con vs *T* = 1.29 ± 1.03 vs 5.39 ± 2.80, *P* = 0.008; UT vs T = 1.29 ± 0.639 vs 5.39 ± 2.80, *P* = 0.007. 14 days: Con vs T = 1.04 ± 0.317 vs 1.47 ± 0.714, *P* = 0.221; UT vs *T* = 1.00 ± 0.658 vs 1.47 ± 0.714, *P* = 0.264. 21 days: Con vs *T* = 1.13 ± 0.623 vs 1.23 ± 0.540, *P* = 0.765; UT vs *T* = 0.579 ± 0.229 vs 1.23 ± 0.540, *P* = 0.022. 28 days: Con vs *T* = 1.12 ± 0.578 vs 1.50 ± 0.467, *P* = 0.244; UT vs *T* = 1.16 ± 0.430 vs 1.50 ± 0.467, *P* = 0.215) (Fig. 4b). Furthermore, Western blotting also validated that the protein level of Nanos2 was significantly increased in the treated side testes of the cryptorchidism group (Fig. 4c).

### SSC homeostasis disrupted in mouse model of cryptorchidism

**Fig. 5:**
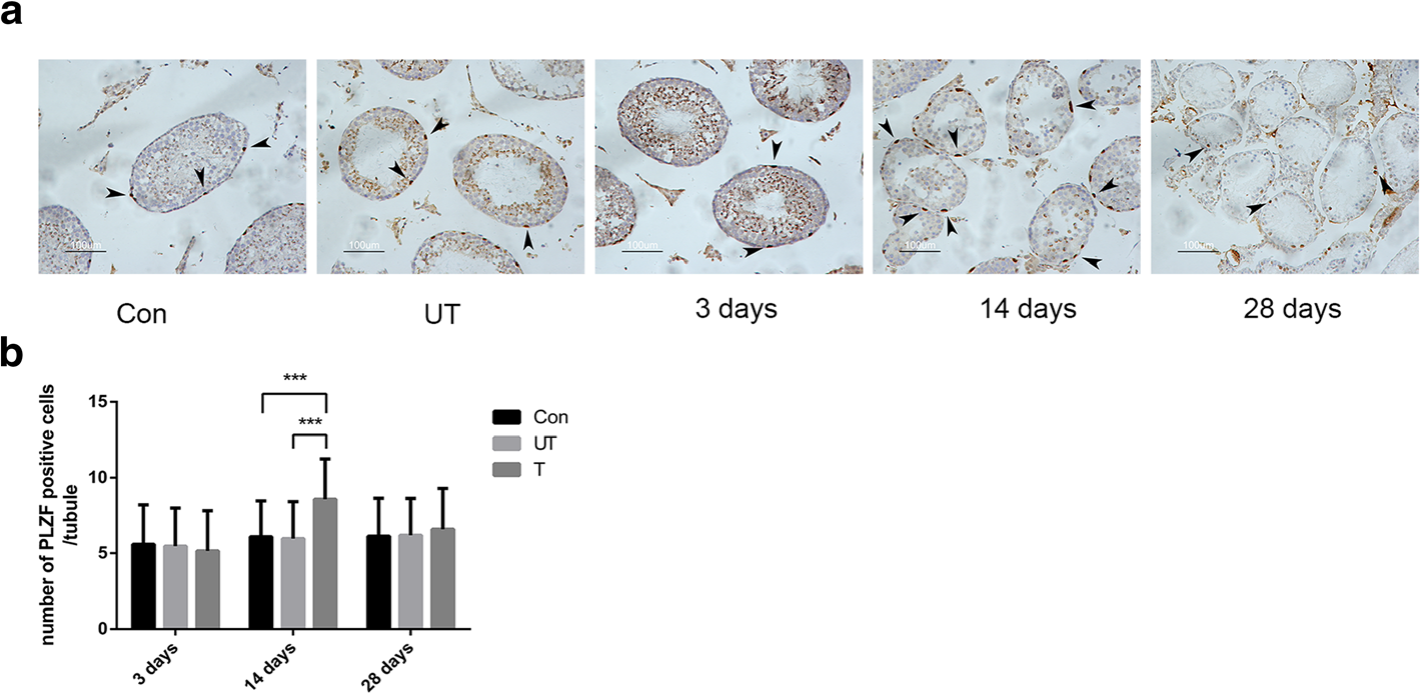
SSC homeostasis was disrupted in mouse model of cryptorchidism. **a** Immunohistochemical staining of testicular tissue with antibody to PLZF. PLZF positive SSCs were indicated by black arrowhead. **b** The number of PLZF positive spermatogonia in treated side testes of cryptorchidism group compared to the untreated side testes and control group. Bar = 100 μm. Con: control; PLZF, promyelocytic leukemia zinc finger; SSCs, spermatogonial stem cells; UT: untreated side; T: treated side. ^***^P < 0.001

SSCs are capable of self-renewal and differentiation because of tissue homeostasis [21]. To investigate whether the abnormal expression of the miR-34c/Nanos2 pathway affects SSC homeostasis in cryptorchid testes, we analyzed the number of PLZF positive spermatogonia in the seminiferous tubules. By counting 80 seminiferous tubules from at least 20 independent microscopic fields, we found that the number of PLZF positive spermatogonia significantly increased in the treated side testes of the cryptorchidism group at 14 days after operation. Interestingly, the PLZF positive spermatogonia in the treated side testes of the cryptorchidism group showed no significant change as compared with the other two groups at 28 days after operation (14 days: *P*_(Con vs T)_ < 0.001; *P*_(UT vs T)_ < 0.001) (Fig. 5a, b).

## Discussion

Spermatogenesis is a complex, highly organized process in which post-transcriptional regulation is indispensable [22]. It is well known that miRNAs are one of the most common agents involved in the post-transcriptional regulation of genes. There are some other miRNAs, such as miR-210, that have been shown to be up-regulated in the testicular tissues of patients with cryptorchidism. miR-210 functions in spermatogenesis by targeting NR1D2 [19]. In the present study, we demonstrated that miR-34c was markedly reduced in the testicular tissues of patients with cryptorchidism. Subsequently, a surgery-induced mouse model of cryptorchidism was established to explore the roles of miR-34c in impaired spermatogenesis of cryptorchidism. Our surgery-induced mouse model of cryptorchidism reveals several key histological features, including morphological abnormalities of seminiferous tubules and an almost absence of mature spermatozoa, which is suitable for research on cryptorchidism. In accordance with the results in humans, miR-34c was significantly down-regulated in the mouse model of cryptorchidism. Recent studies have reported that miR-34c is involved in the proliferation, apoptosis, invasion, and prognosis of multiple malignancies. Here, we propose that miR-34c may serve as a novel target for treatment of infertility caused by cryptorchidism.

Nanos2 is a germ cell-specific gene that encodes an evolutionarily conserved RNA binding protein. In the embryonic period, Nanos2 promotes the development of male primordial germ cells (PGCs) and inhibits them from meiosis [23, 24]. In the process of spermatogenesis of testes, Nanos2 suppresses the differentiation of SSCs and maintains the stability of the SSC pool [7]. Knockout of Nanos2 causes the apoptosis of male PGCs, eventually leading to the loss of testicular germ cells and infertility of male mice [25]. Interestingly, the overexpression of Nanos2 resulted in an increase in the number of PLZF positive spermatogonia by blocking the differentiation of SSCs rather than hyperproliferation or a decrease of apoptosis [8]. In the present study, we found that the mRNA level of Nanos2 showed no statistical difference between the patients with cryptorchidism and the controls, maybe due to the lack of sufficient samples. In contrast to miR-34c, the protein level of Nanos2 was significantly up-regulated in the cryptorchidism model. The mRNA level of Nanos2 only increased at some time points in the model. We cannot rule out the possibility that the mRNA of Nanos2 is stable with a long half-life time owing to its complex secondary structure. Generally, we found that miR-34c and Nanos2 showed a negatively correlated expression pattern in the mouse model. However, we can’t rule out the heat-sensitive expression of Nanos2 itself. There is a possibility that Nanos2 was sensitive to heat stress and directly up-regulated in cryptorchid testes, rather than involving miR-34c.

Stress granules (SGs) are conserved cytoplasmic particles that store non-translated messenger ribonucleoproteins (mRNPs) formed under stress conditions, such as heat stress, hypoxia or insufficient nutrition supply [26–28]. In a previous study, the formation of SGs were observed in spermatogonia in the testes of mice incubated at 42 °C for 20 mins [29]. Nanos2 is one of the important components of SGs and possesses the role of promoting mRNP assembly [7, 30]. In the present study, the surgery-induced mouse model of cryptorchidism, with the abnormal expression of miR-34c/Nanos2, emerged as a disruptor of SSC homeostasis. We propose that on the one hand up-regulated Nanos2 is involved in the formation of SGs to prevent the apoptosis of SSCs induced by heat stress. On the other hand, up-regulated Nanos2 disrupts the balance between SSC self-renewal and differentiation and eventually damages the spermatogenesis of cryptorchid testes.

## Conclusions

In the present study, we demonstrated that miR-34c was significantly decreased in testicular tissues of patients with cryptorchidism. In contrast to miR-34c, the protein level of Nanos2 was significantly up-regulated in the mouse model of cryptorchidism. The abnormal expression of miR-34c/Nanos2 disrupted the balance between the self-renewal and differentiation of SSCs, eventually damaging the spermatogenesis of cryptorchid testes. Our research provides a new insight for the mechanism of male infertility caused by cryptorchidism. We propose that miR-34c may serve as a novel target for treatment of infertility caused by cryptorchidism.

## Abbreviations

Con: Control
Cry: Cryptorchidism
GAPDH: Glyceraldehyde-3-phosphate dehydrogenase
miRNAs: microRNAs
mRNPs: Messenger ribonucleoproteins
NC: Negative control
OA: Obstructive azoospermia
PGCs: Primordial germ cells
PLZF: Promyelocytic leukemia zinc finger
SGs: Stress granules
SSC: Spermatogonial stem cell
T: Treated side
UT: Untreated side
